# Effects of Dietary Chromium Supplementation During Late Lactation on Productive Performance, Milk Composition, and Immune and Antioxidant Responses in Dairy Cows

**DOI:** 10.3390/ani15213111

**Published:** 2025-10-27

**Authors:** Natália Turcatto, Guilherme Luiz Deolindo, Maksuel Gatto de Vitt, Maisa Damo, João Gustavo Weschenfelder Wandscheer, Daiane Manica, Gilnei Bruno da Silva, Margarete Dulce Bagatini, Aleksandro Schafer Da Silva

**Affiliations:** 1 Departamento de Zootecnia, Universidade do Estado de Santa Catarina, Chapecó 89815-630, SC, Braziljgw.wandscheer@edu.udesc.br (J.G.W.W.); 2 Programa de Pós-Graduação em Bioquímica e Biologia Molecular, Universidade do Estado de Santa Catarina, Lages 88520-000, SC, Brazil; 3 Programa de Pós-Graduação em Zootecnia, Universidade do Estado de Santa Catarina, Chapecó 89815-630, SC, Brazil; 4 Centro de Ciências da Saúde, Universidade Federal Fronteira Sul, Chapecó 89815-899, SC, Brazil

**Keywords:** cow, chromium, immune system, antioxidant, milk production

## Abstract

The study evaluated whether organic chromium supplementation in the diets of Jersey cows in the final third of lactation has positive effects on productive performance and milk quality, as well as markers of metabolism, hematology, immunological, and oxidative response. We used 22 cows in a 56-day study, divided into two groups: control (n = 11) and chromium treatment at 10 mg/kg of dry matter per day (n = 11). We evaluated these animals’ productive performance markers (milk production, feed intake, and lactation persistence), milk quality (composition and somatic cell counts), and blood biomarkers to assess animal health. Chromium concentration at the beginning of the experiment was within normal limits for the animal category (221 to 246 nM). The cows’ consumption of organic chromium increased its bioavailability in the body, with higher concentration in the serum (*p* = 0.01) and milk (*p* = 0.013) of Jersey cows compared to the control cows. Supplementing cows with chromium resulted in higher fat-corrected milk production (*p* = 0.05), longer lactation persistence (*p* = 0.05), and feed efficiency (*p* = 0.02) compared to the control group. There was a lower SCC (*p* = 0.01) on days 28, 42, and 56, as well as a higher percentage of fat in the milk of supplemented cows on days 28 and 42 compared to that of the control group (*p* = 0.01). As a treatment effect (*p* ≤ 0.05), we found cows supplemented with chromium had a higher concentration of insulin, immunoglobulin G, and creatine kinase activity dismutase compared to control animals. Considering the treatment × day interaction (*p* ≤ 0.05), we observed greater activity of the creatine kinase enzymes (days 14, 28, and 56), as well as lower cholinesterase activity (days 42 and 56) compared to those of the control. The concentration of globulins (*p* = 0.05) and immunoglobulin G (*p* = 0.01) on day 56 was higher in cows that consumed chromium. Higher superoxide dismutase activity on days 42 and 56 (*p* = 0.04) was observed in the blood of cows supplemented with organic chromium compared to that of the control. Therefore, we conclude that the addition of organic chromium was beneficial to maintaining lactation persistence and increasing fat-corrected milk production.

## Simple Summary

Chromium supplementation increased lactation persistence and increased fat-corrected milk production. Over the course of chromium consumption, we observed an effect on milk quality, notably increased milk fat, and reduced somatic cell count. Chromium supplementation in cows has nutraceutical potential, increasing the concentration of this mineral in milk. Cow health was improved because of the stimulation of the humoral immune response, associated with anti-inflammatory and antioxidant effects.

## 1. Introduction

Animals in the final third of lactation need to maintain high production while simultaneously maintaining fetal development [1]. During this period, the immune response tends to decline, making animals even more susceptible to health challenges, such as increased somatic cell count (SCC) because of the presence of microorganisms within the mammary gland, which can result in mastitis [2]. Furthermore, as pregnancy progresses, oxidative reactions resulting from cellular respiration can lead to physiological oxidative stress [3]. Several studies indicate that the use of minerals at a supra-nutritional concentration plays a role in increasing gains and reducing production costs in dairy cattle farming. These additives, which can be in inorganic or organic forms, aid performance, increase feed efficiency, and improve reproduction [4]. The mineral chromium in recent years has shown benefits when fed to lactating cows [5,6], but updated cow diet formulation guidelines still lack a recommendation for chromium [7].

Cr can be an excellent supplementation strategy for late-lactation cows due to its efficient immune and antioxidant response, minimizing the negative consequences related to environmental challenges [8]. Furthermore, it increases dry matter intake (DMI), increases milk production in early lactation [9], increases glucose and insulin concentration, and decreases in non-esterified fatty acids (NEFAs) [10]. Regarding milk composition, research shows no change in total-solid content when cows were supplemented with chromium [8]. 

The main function of chromium in animal nutrition is related to its effect on insulin, which influences the metabolism of carbohydrates, lipids, and proteins [11]. Specifically, chromium enhances the action of insulin in cells, facilitating the interaction between insulin and receptors in muscle and adipose tissue, allowing glucose to enter cells to be used as energy or stored. In pre- and postpartum phases, chromium supplementation is even more important because of the physical and metabolic stress caused by these periods [12,13]. It is known that stress causes increased glucose expenditure to meet energy demands, resulting in the increased release of cortisol, a hormone that resists the action of insulin and leads to increased urinary chromium excretion, resulting in accelerated glucose metabolism [14]. Increased circulating cortisol and low insulin concentration can lead to increased fat mobilization in the body, increasing the risk of stress-related diseases, particularly mastitis, milk fever, and ketosis [15]. However, the available results on chromium supplementation in cows in the final third of lactation are scarce, but it is believed that this mineral has the potential to improve immune and antioxidant responses, promoting productivity.

As mentioned above, studies have shown that adding chromium to cows’ diets has resulted in health benefits, but NASEN [7] does not yet have chromium recommendations for any production phase. Studies like this could support researchers, so that chromium can be included in the next edition of NASEN, reporting its nutritional requirements for cows. In the final third of lactation, the main justification for its use is related to its multifactorial effects, including those related to the insulin pathway, which can stimulate the mobilization of body fat for milk production, preventing the cow from increasing her body condition score, which can cause problems in the next lactation. Therefore, the objective of this study was to evaluate whether organic chromium (bound to yeast) supplementation in the diet of Jersey cows in the final phase of lactation has positive effects on the modulation of metabolism, immune, inflammatory, and oxidative responses, as well as on the persistence of lactation.

## 2. Materials and Methods

The research took place at the Experimental Farm of the Higher Education Center of the West of the Santa Catarina State University in Guatambu, Santa Catarina, Brazil. The experimental protocol was approved on 30 August 2024 by the Ethics Committee on Animal Use of the Santa Catarina State University, No. 6332260824.

### 2.1. Mineral

The mineral used was organic chromium (Cr^3+^), a commercial product of Yessinergy/Olmix (Elements Chromium^®^, Campinas, SP, Brazil). The company’s chromium is composed of yeast chromium, so at 1%, it represents 10,000.00 mg/kg of yeast chromium. Chromium was added to the concentrate supplied individually, using individual feeders, where the animal was contained by stanchion twice a day, as detailed below.

### 2.2. Animals and Installation

Twenty-two multiparous Jersey cows in the final third of lactation (270 days in lactation) were used in this study. The animals, housed in a compost barn, were milked two to four times daily using a free-range milking system using a DeLaval VMS™ V300 robotic milker (Delaval do Brasil, Jaguariúna, São Paulo, Brazil). The facility featured fans distributed throughout the bedding and feeding research areas, as well as a manually activated sprinkler system for thermal control in the feeder line. To ensure animal welfare, the animals had free access to a rotating cow brush (DeLaval SCB). Historically, these cows were already in the compost barn system since calving, accustomed to the milking and feeding system. Before starting the experiment, the animals received recombinant bovine somatotropin (BST-r) (Boostin, MSD Animal Health, Rahway, NJ, USA) at 12-day intervals. This hormone was applied to the cows at the base of the tail for 100 days before the experiment, characterizing the period between 150 and 250 days of lactation.

### 2.3. Experimental Design and Diet

The experiment lasted 56 days, with the initial 14 days considered an adaptation period. The design was completely randomized, with two treatments and 11 replicates per group. Cows were divided according to milk production, gestation number, and days in lactation (DIL): control (n = 11)—no supplementation; chromium treatment (n = 11)—supra-nutritional chromium supplementation of 10 mg chromium/kg dry matter intake.

The basal diet was formulated to meet nutritional requirements based on NASEM [7], with the characteristics of the animals used for the formulation shown in Table S1. The chromium group received supra-nutritional supplementation of the concentrate with the organic mineral chromium. The supplementation concentration used for the chromium group was based on a study by McNamara and Valdez [9], who observed better results with supplementation of 10 mg of chromium propionate/kg of dry matter (DM), because of an increase in dry matter intake (DMI), as well as increased milk production and better use of glucose by the cells.

Cows were fed three times a day. The first two feedings were conducted in individual feeders where the cows were restrained by a cage, with feeding beginning at 7:40 am and 3:00 pm, respectively. Cows remained restrained at their feeders for approximately 1.5 h or until feeding was completed. The third feeding was distributed in six automatic smart feeders (Intergado^®^, Força, Betim, MG, Brazil), released to the animals after 6:00 pm, maintaining free consumption during the night, but with all feed intake recorded. Additionally, during milking, the animals had access to up to 2 kg/day of pelleted concentrate specifically for use in robotic milking (Nutrialfa^®^ Bovino Robô AP, Chapecó, SC, Brazil). The proximate composition of the diets provided in the individual feeders is shown in Table S2.

### 2.4. Data and Sample Collection

On days 1, 14, 28, 42, and 56, blood samples were collected from the coccygeal vein, and individual milk samples were collected. The blood samples were stored at a temperature below 5 °C in a cooler until they arrived at the laboratory. A blood collection tube (Vacuplast^®^, Pardis, Vespasiano, MG, Brazil) was used with needles and a vacuum tube. Three tubes were collected per animal: one of a 4 mL tube containing anticoagulant (EDTA K3) for a complete blood count, and another containing sodium citrate for superoxide dismutase (SOD) analysis, as well as a 10 mL tube containing clot activator for serum separation. The complete blood count was performed immediately upon arrival at the laboratory, as was serum separation. The tubes were centrifuged (QUIMIS^®^, model Q222T, Diadema, SP, Brazil) at 1580× *g* for 10 min at room temperature. The separated samples were stored in an Eppendorf at −20 °C until analysis. Milk collection, individualized for each animal, was performed in specific tubes using the robot’s own collector, which collects a homogeneous sample of the entire cow’s milking. A Brononata^®^ (Larboclin, Pinhais, PR, Brazil) preservative tablet was added to the samples, and the milk samples were stored at room temperature until arrival at the laboratory.

Samples of concentrate, silage, hay, pelleted concentrate, and the total basal diet were collected during the experiment (days 10, 20, 30, 40, and 50) and kept frozen until the end of the study. The samples were thawed and homogenized to obtain a representative sample of the experimental period. Pre-drying began in an oven with forced air circulation at 55 °C for 72 h, as well as subsequent grinding in a Wiley mill (Marconi^®^, model MA340, Piracicaba, SP, Brazil) using a 1 mm mesh sieve.

### 2.5. Feed Composition Analysis

Dry matter content was determined after the samples were ground and stored for 24 h in a forced-ventilation oven at 105 °C. To quantify the mineral matter, the samples were placed in a muffle furnace at 600 °C until the organic matter was completely burned [16]. Crude protein content was determined by micro-Kjeldahl using the official method AOAC 2001.11 [17]. Ether extract quantification was performed in an automatic fat extractor (VELP Scientifica^®^, model SER 158, Shanghai, China), following the official method-AOAC 2003.05 [17], and replacing diethyl ether with petroleum ether. The composition of silage, hay, concentrate, and pelleted concentrate supplied by the milking robot can be found in Table 1.

### 2.6. Hemogram

The blood count was performed on an automatic hematology analyzer (EQUIP VET^®^ 3000, Barueri, SP, Brazil), focusing on the erythrocyte count (×10^6^/µL), total leukocyte count (differentiating lymphocytes, granulocytes, and monocytes), and platelets (×10^3^/µL), as well as the hemoglobin concentration (mg/dL) and hematocrit percentage (%).

### 2.7. Serum Biochemistry

Serum biochemistry was performed using an automatic analyzer (Zybio^®^ EXC 200, Barueri, SP, Brazil) and commercial kits (Analisa^®^, Belo Horizonte, Brazil). The variables analyzed were the activity of the enzymes creatine kinase and cholinesterase (U/L), as well as analyses of total protein (g/dL), glucose (mg/dL), albumin (g/dL), cholesterol (mg/dL), C-reactive protein (mg/dL), urea (g/dL), ferritin (µg/L), immunoglobulin G and A (mg/dL), and triglycerides (mg/dL). Serum insulin (µU/mL) concentration was determined by enzyme-linked immunosorbent assay (ELISA) using a commercial BovineInsulin Elisa Kit (USCN Life Science^®^, Houston, TX, USA).

### 2.8. Oxidative Status

The oxidative status variables evaluated in blood serum were reactive oxygen species (ROS), thiobarbituric acid-reactive substances (TBARSs), and myeloperoxidase (MPO) activity; SOD activity was measured in whole blood. All analyses were performed in triplicate using specific biochemical methodologies described below. ROS formation was estimated by the fluorimetric protocol established by Ali et al. [18], in which a 10 µL volume of serum was incubated with the same amount of 2′,7′-dichlorofluorescein diacetate (DCFH-DA, 7 µM) and 240 µL of sulfate-buffered saline (PBS). The quantification of TBARSs in serum followed the methodology described by Jentzsch et al. [19], a reaction with thiobarbituric acid (TBA) in the presence of MDA results in a pink product that can be read at 532 nm. MPO activity was analyzed using a modified peroxidase system in the presence of H_2_O_2_ as an oxidizing agent. MPO catalyzes the oxidative bond of phenol and 4-aminoantipyrine (AAP), yielding a colored product, quinoneimine, with a maximum absorbance of 492 nm [20]. SOD activity was determined based on the inhibition of the reaction of the superoxide radical with adrenaline, as described by McCord and Fridovich [21].

### 2.9. Chromium Concentration in Feed, Milk, and Blood Serum

Basal diet sample preparation (concentrate, corn silage, and hay) was performed by microwave-assisted wet decomposition (MAWD) using an Ethos Easy™ decomposition system (Milestone™, Sorisole, Italy) equipped with a rotor with 44 PTFE-TFM containers with an internal capacity of 100 mL (MAXI-44 rotor, Milestone™). Decomposition and analysis of feed samples were performed in triplicate. Approximately 250 mg of the sample was weighed directly into the decomposition containers following the methodology described by Klein et al. [22].

The measurement of chromium concentration in serum (days 1 and 56) and milk (day 56) followed the methodology described by Glombowsky et al. [23]. Chromium concentration was initially determined by digesting the sample with the addition of nitric, acetic, and perchloric acids, followed by constant heating. After this process, hydrochloric acid was added, and chromium concentration was subsequently determined by mass spectrometry (Thermo Fisher Scientific Inc., São Paulo, Brazil). The results are expressed in nM.

### 2.10. Milk Quality

Milk quality analyses were performed by the Centralized Milk Analysis Laboratory of the Paraná Dairy Herd Analysis Program, accredited by the Ministry of Agriculture, Livestock and Supply. The total protein, lactose, fat, total solids, and milk urea nitrogen (MUN) contents, expressed as percentages, were determined by Fourier transform mid-infrared spectrometry according to ISO 9622/IDF 141:2013, describe by Klein et al. [22]. The somatic cell count (SCC) was expressed as ×10^3^/mL and was determined by flow cytometry according to ISO 13366-2/IDF 148-2:2006, describe by Klein et al. [22].

### 2.11. Productive Performance

Daily milk production was obtained from the robotic milking system, using average production data from the last seven days. Since milking was free-access, cows differed in the number of milkings per day, with some cows milked twice a day and others milked three to four times a day. Dry matter intake (DMI) was calculated by adding the partial diet consumed in individual feeders during the day, in automatic feeders at night, and the pelleted concentrate fed by the robot during milking. Knowing the moisture content of these feeds, intake was converted to DMI based on the results obtained from the feed analyses. Feed efficiency was obtained by calculating milk production/DMI. Furthermore, lactation persistence was obtained by the equation:

$$Lactation\;Persistence\;(\%)\;=\;\{1\;-\;[\frac{(PL_{i}-PL_{f})\times 30}{(f-i)\times PL_{i}}]\}\times 100$$

in which *PL_i_* is the initial milk production, *PL_f_* is the final milk production, *i* is the initial day (day zero), and *f* is the final day, i.e., day 56.

### 2.12. Statistical Analyses

Data were analyzed using the MIXED procedure in SAS^®^ (SAS Inst. Inc., Cary, NC, USA; version 9.4) with Satterthwaite approximation to determine the denominator (degrees of freedom) for the fixed-effects test. Milk production, lactation persistence, and feed efficiency were tested for treatment fixed effects, using animals (treatment) as random effects. Daily milk production data, as well as all blood and milk parameter results, were analyzed as repeated measures and were tested for treatment fixed effects, daily fixed effects, and treatment x day interaction, using animals (treatment) as random effects. Day 1 results were included as an independent covariate. The first-order autoregressive covariance structure was selected according to the Akaike least information criterion. Means were compared using the PDIFF method, and results were expressed as least squares means (LSMEANS) followed by the standard error of the mean (SEM). Significance was defined when *p* ≤ 0.05 and trend when *p* > 0.05 and ≤ 0.10.

## 3. Results

### 3.1. Chromium Concentration in Milk and Serum

**Figure 1:**
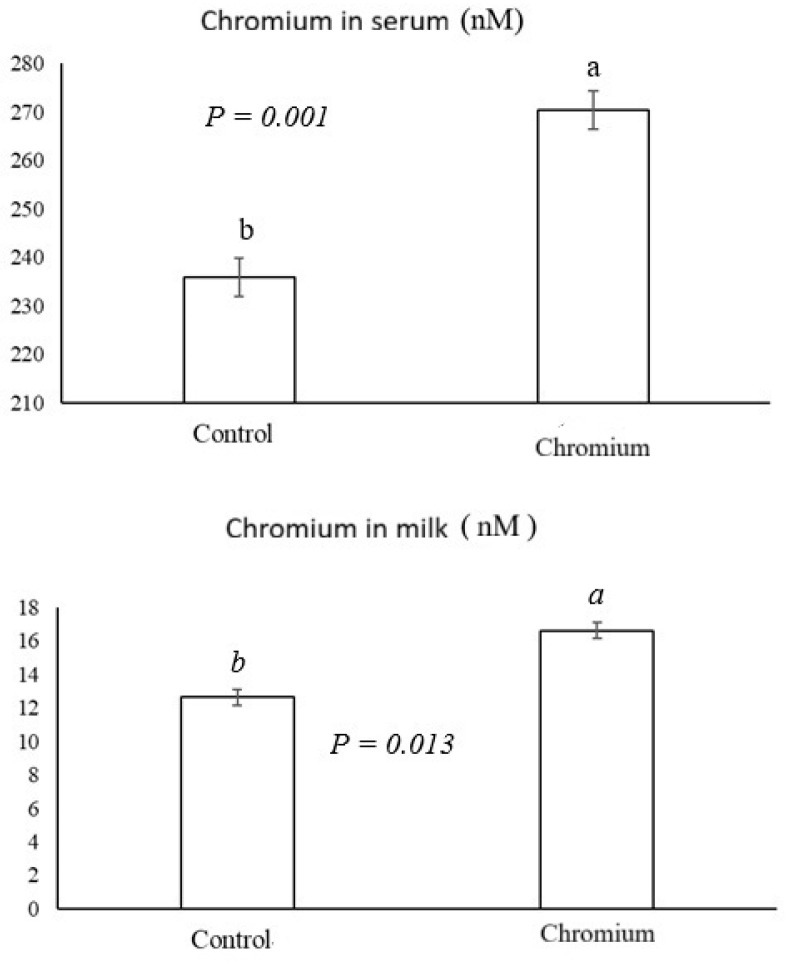
Chromium concentration in serum and milk of cows supplemented with chromium on day 56 of the experiment. Different letters (a,b) per bar differ statistically from each other in each variable.

In the basal diet, chromium concertation was 4.54 and 13.9 mg per kg of DM in the control and chromium groups, respectively. Serum chromium concentration on day 1 in cows ranged from 221 to 246 nM. The results for chromium concentration in serum and milk on day 56 are presented in Figure 1. Chromium concentration in serum and milk was higher in the group of cows supplemented with chromium compared to the control group (*p* = 0.001 and *p* = 0.013, respectively).

### 3.2. Productive Performance

The results of productive performance are shown in Table 2. Milk production did not differ between groups (*p* = 0.26). Greater persistence of lactation was observed in the chromium-treated group compared to that of the control group (*p* = 0.05). Furthermore, the feed efficiency of the treated group was also higher than that of the control group (*p* = 0.02). It is noted that milk production when corrected for fat percentage was higher in the treated group compared to that of the control group (*p* = 0.05).

### 3.3. Milk Quality

Milk composition and quality results are presented in Table 3. It was observed that the fat percentage on days 28 and 42 was higher in the chromium-treated group compared to that of the control group. The SCC count was lower in the chromium-supplemented cows on days 28, 42, and 56 compared to that of the control group. A day effect was observed in both the treated and control groups, reducing throughout the experiment in the milk of cows that consumed chromium, unlike that in the control group, where there was an increase in SCC count in the cows. The treatment × day interaction occurred, with lower SCC counts being observed in the treated-group cows compared to that in the control group on days 28, 42, and 56 of the experiment.

### 3.4. Hemogram and Biochemistry

There was no statistical difference (*p* > 0.01) between the groups for the number of erythrocytes, total leukocytes, lymphocytes, monocytes, granulocytes, and platelets, as well as hemoglobin concentration and hematocrit percentage (Table 4).

**Figure 2:**
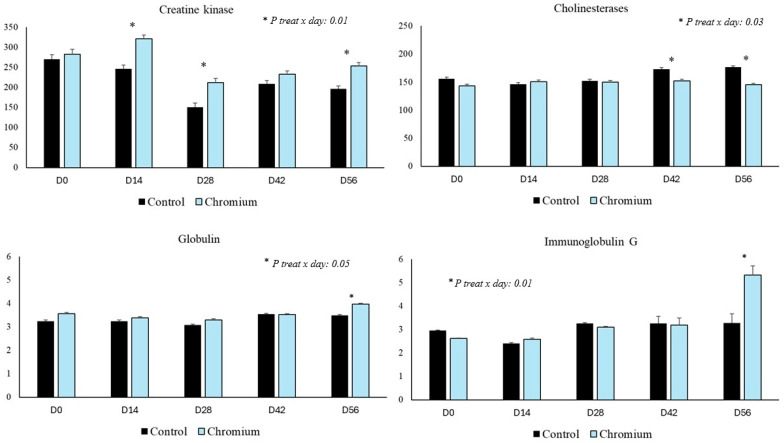
Serum activity of creatine kinase and cholinesterase variables, as well as globulin and immunoglobulin G concentration at specific times of the experiment.

Serum biochemistry results are also presented in Table 4 and Figure 2. A treatment × day interaction was observed in creatine kinase (CK), cholinesterase, and immunoglobulin G (IgG) (*p* < 0.05), as well as a treatment effect for these variables, with the exception of globulins, where there was only a trend (*p* = 0.06). CK activity was higher in the group treated with chromium on days 14, 28, and 56 compared to that in the control group. Globulin and IgG concentrations were higher in the serum of cows supplemented with chromium compared to those in the control group only on day 42. Cholinesterase activity in cows in the treated group was lower on days 42 and 56 compared to that in the control. There was a treatment effect on serum insulin concentration, being higher in cows in the treated group compared to those in the control group. Serum insulin was higher in the chromium-supplemented group when compared to that in the control group (*p* < 0.05). There was a trend (*p* = 0.06) for higher glucose concentration in cows supplemented with chromium (Table 4). No treatment effect or treatment × day interaction was observed for the variables albumin, total cholesterol, total protein, ferritin, C-reactive protein, triglycerides, urea, and immunoglobulin A (Table 4).

### 3.5. Oxidative Status

Oxidative status results are presented in Table 5. No treatment × day interaction or treatment effect was observed for ROS and TBARS concentration, as well as MPO activity. However, there was a treatment × day interaction for SOD activity, which was higher in the chromium-supplemented group when compared to that in the control group on days 42 and 56 (*p* = 0.04).

## 4. Discussion

The mineral chromium plays a key role in metabolism, primarily by modulating insulin in organisms. It regulates glucose entry into cells, especially in muscle and adipose tissue. This mineral stimulates insulin receptor efficiency, improving insulin sensitivity and consequently improving glucose utilization [24]. The study found that insulin concentration was higher in the treatment group, resulting from chromium’s metabolic modulation. The study by Qiang et al. [25] demonstrated similar results, observing increases in insulin and glucose with chromium picolinate supplementation at concentrations up to 10.8 mg/day.

Chromium supplementation in dairy cows positively influences chromium concentrations in milk and serum, increasing chromium bioavailability in the cow’s body. The study by Giometti et al. [26], using a concentration of up to 2.38 mg/animal/day of yeast complexed with chromium, demonstrated an effect of serum chromium concentration. However, lower doses of 0.59 and 1.19 mg/animal/day demonstrated no effect. In contrast, the study by Wang et al. [14], when supplementing doses of up to 12 mg/animal/day of chromium propionate, found no changes in milk or urine concentrations. Results were similar to those of the study by Lloyd et al. [27], which found no effect on milk chromium concentrations with a supplementation of 2 mg of chromium propionate/kg DM. These studies suggest that the organic form of the mineral, such as chromium yeast, has greater bioavailability in the body because of its increased concentration in serum and milk, thus being more effective at increasing serum concentrations. From a nutraceutical perspective, the greater presence of chromium in milk may be beneficial to consumers, given its functions in the mammalian body.

In addition to increasing serum and milk chromium concentrations, chromium supplementation increased lactation persistence without altering dry matter intake. According to the literature, the increased lactation persistence would be related in most cases to increased DMI, resulting in a decrease in free fatty acids in the blood and greater glucose utilization through the action of insulin [9], consequently increasing milk production, according to a meta-analysis by Malik et al. [28]. According to these researchers, cows supplemented with chromium increased DM intake by up to 0.72 kg/day (which did not occur in the present study) and with an additional production gain of up to 1.20 kg/day. Smith et al. [29] found precisely this, as there was an increase in DMI and milk production as chromium doses increased, with doses of 0.003 or 0.06 mg of chromium-bound methionine/kg of metabolic body weight standing out. Wu et al. [30] also observed an increase in milk production, but without an increase in DMI. According to the authors, this can be explained by the synthesis of lactose, as it maintains osmotic pressure, resulting in greater milk production because of the increased lactose production. As we found that chromium supplementation increased insulin concentration, and knowing that this mineral enhances the action of insulin, facilitating the entry of glucose into cells, which is used as energy or stored in the form of glycogen, it can improve the metabolism of carbohydrates, lipids, and proteins. Chromium helps with better energy utilization and fat mobilization, a mechanism similar to the application of bovine somatotropin, and a practice that was performed prior to the experiment with these cows here.

SCC is an important inflammatory response tool in the mammary glands, reflecting on the health of these glands. In this study, chromium supplementation decreased SCC, which may also contribute to increased milk production. This may be explained by the fact that chromium improves immune responses. A study by Andrei et al. [31] demonstrated that SCC is also related to oxidative stress, and in this case, chromium supplementation improves these parameters, in addition to immunological ones. The increase in milk fat can be explained by the reduced mobilization of fat by the body, thus resulting in greater energy availability for milk fat synthesis [32]. Research by Hayirli et al. [33] found no changes in protein synthesis, because chromium acts primarily on energy and lipid metabolism via insulin, while protein synthesis is linked to amino acid metabolism, in which chromium does not act directly. However, in the 1990s, researchers [34] reported that chromium supplementation did not alter fat, lactose, or protein parameters in milk, unlike what occurred in this study. Considering that recent years have seen significant innovations and technologies in animal feed formulations, these techniques may have made chromium more bioavailable, thus influencing the fat profile of milk. We believe that there was an indirect effect of chromium supplementation on the health of the cows, and this resulted in a reduction in SCC due to the positive immunological effect (IgG), as well as an increase in milk fat content through the modulation of lipid metabolism. Thus, chromium supplementation demonstrates nutraceutical potential, including improved metabolic balance, reduced oxidative stress, and superior milk quality, all characteristics sought by consumers [8].

Chromium consumption has a direct impact on metabolic health, as can be seen in the results of CK, an enzyme involved in energy metabolism [35]. Furthermore, the increase in CK activity is likely related to the metabolic system’s response to organic chromium consumption. The reduction in cholinesterase activity may have an anti-inflammatory role, because lower enzyme activity results in less acetylcholine being degraded, an anti-inflammatory molecule [36]. However, the pathway of chromium action on the cholinergic system is unknown, and it may have a direct or indirect effect, which is related to improved cow health.

The increase in globulin and IgG concentration is both related to the animals’ immune and/or inflammatory response. Increased globulins may be an indicator of inflammatory processes, due to the rapid increase in acute phase proteins in the blood, which can be modulated by stress factors at the end of gestation [37]. In this study, the increase in IgG was observed only on day 56, the final phase of gestation. Therefore, this increase may be related to immunological preparation for parturition and the passive transfer of immunity to the calf, according to the literature [38]. Similar results were observed by Yujuan Li et al. [39], who reported an increase in IgA, IgM, and IgG concentrates in the colostrum and blood of kids supplemented with 0.4 mg of chromium per animal. The literature has already described that chromium supplementation has a strong importance for immune function, being able to improve animal health [40]. Therefore, these increases in globulins, such as IgG, represent a positive response in the cow.

In the experiment, the parameters related to oxidative stress, such as ROS, TBARSs, and MPO enzyme activity, did not show significant changes between the groups evaluated. These results suggest that organic chromium supplementation does not always alter oxidative biomarkers, but in this case, it stimulated the activity of the antioxidant enzyme SOD. Shan et al. [41] in their studies, supplementing chromium yeast at a concentration of 0.11 mg/kg DM, improved the oxidative status of cows under heat stress, a condition directly associated with oxidative stress. Therefore, the absence of changes in oxidative markers, but the elevation of SOD in the group of cows supplemented with chromium, is more indicative of the strong participation of chromium in the antioxidant action of the animals [40], even if the cow is not in a challenging condition. This suggests that supplementation maintains cellular integrity and improves the physiological response, which contributed to greater lactation persistence under environmental conditions suitable for the animals (without heat stress).

## 5. Conclusions

Chromium supplementation at a dose of 10 mg/kg of dry matter intake increased serum and milk chromium concentrations, thus increasing the bioavailability of this mineral. Higher circulating chromium concentrations demonstrated an increase in bioavailability as well as an increase in insulin concentration. Creatine kinase activity as well as the concentration of globulins and immunoglobulin G were higher in supplemented cows. The activity of the antioxidant enzyme superoxide dismutase increased in cows supplemented with chromium. Chromium intake by cows resulted in greater lactation persistence and higher fat-corrected milk production. It also demonstrated improvements in milk quality, notably with increased milk fat and decreased SCC, indicating a decrease in mammary gland inflammation.

## Figures and Tables

**Table 1:** Chemical composition of silage, concentrate, hay, and pelleted concentrate.

Variables	Silage	Hay	Concentrate	Pelleted Concentrate
Dry matter—DM (%)	33.67	81.83	89.05	89.15
Mineral matter % DM	6.64	9.21	10.07	7.43
Crude protein % DM	3.89	7.29	24.26	19.93
Ether extract % DM	3.21	1.94	3.83	3.98
NDF, % DM	59.7	69.8	29.9	-

**Table 2:** Productive performance of Jersey cows supplemented with organic chromium.

Variables	Control	Chromium	SEM	*p*-Value
Milk production, kg				
D −30 to 1 (pre-experiment)	24.41	23.69	0.82	0.73
D 1 to 56 (experimental period)	18.95	20.00	0.71	0.26
Fat-corrected milk (4%FCM) ^1^ kg	19.56 ^b^	21.71 ^a^	0.48	0.05
Persistence of lactation (%)	77.66 ^b^	84.42 ^a^	2.26	0.05
Daily dry matter intake, kg	16.13	16.27	0.42	0.97
Feed efficiency (kg/kg)	1.17 ^b^	1.23 ^a^	0.01	0.02

^1^ Milk production corrected to 4% fat (4%FCM) was estimated by the equation proposed by the NASEN [7]: FCM = 0.4 × (kg milk produced) + 0.15 × (% fat) × (kg milk produced). Persistence (%) = [1 − ((Previous Volume − Current Volume) − Previous Volume) × (days/interval)] × 100. Different letters (a,b) per line differ statistically from each other in each variable.

**Table 3:** Composition and quality of milk from Jersey cows supplemented with organic chromium.

Variables	Control	Chromium	SEM	*p*: Treat ^1^	*p*: Day ^2^	*p*: Treat × Day ^3^
Fat (%) ^4^	4.21 ^b^	4.57 ^a^	0.14	0.04	0.31	0.01
Protein (%) ^4^	3.77	3.84	0.10	0.49	0.89	0.37
Lactose (%) ^4^	4.40	4.47	0.08	0.54	0.75	0.66
Total solids (%) ^4^	13.42	13.81	0.19	0.23	0.34	0.15
Urea (mg/dL) ^4^	14.05	15.07	1.01	0.78	0.25	0.81
SCC (×10^3^ mL) ^4^	363 ^a^	115 ^b^	38.4	0.03	0.01	0.01
Fat (%) ^2,3^						
d1	4.48	4.49	0.28			
d14	4.08	4.38	0.31			
d28	4.23 ^b^	4.60 ^a^	0.16			
d42	4.27 ^b^	4.81 ^a^	0.23			
d56	4.26	4.48	0.14			
SCC (×10^3^/mL) ^2,3^						
d1	255 ^C^	255 ^A^	36.4			
d14	320 ^B^	211 ^AB^	47.9			
d28	521 ^aA^	185 ^bB^	52.4			
d42	336 ^aB^	90.9 ^aC^	40.2			
d56	274 ^aC^	95.1 ^aC^	41.5			

^1^ Treatment effect when *p* < 0.05, with the difference between groups illustrated by different lowercase letters (a, b) in the same row. ^2^ Day effect when *p* < 0.05, illustrated by uppercase letters (A, B, C) in the same column for milk fat in both groups. ^3^ The table demonstrates the treatment versus day interaction for fat on days 1, 14, 28, 42, and 56 of the experiment, with lowercase letters (a, b) in the same row. ^4^ Corresponds to the effect of the treatment, represented by the average of the experimental period of 56 days.

**Table 4:** Hemogram and serum biochemistry of Jersey cows supplemented with organic chromium.

Variables	Control	Chromium	SEM	*p*: Treat ^1^	*p*: Day ^2^	*p*: Treat × Day ^2^
Total leukocytes (× 10^3^ μL) ^3^	6.84	6.83	0.31	0.94	0.87	0.92
Lymphocyte (×10^3^ μL) ^3^	3.87	3.81	0.18	0.92	0.90	0.82
Granulocytes (×10^3^ μL) ^3^	2.13	2.18	0.15	0.89	0.91	0.95
Monocyte (×10^3^ μL) ^3^	0.83	0.82	0.08	0.93	0.90	0.91
Erythrocytes (×10^6^ μL) ^3^	5.19	5.07	0.02	0.71	0.65	0.76
Hemoglobin (g/dL) ^3^	9.12	9.07	0.11	0.84	0.78	0.87
Hematocrit (%) ^3^	25.6	25.4	0.35	0.94	0.95	0.92
Platelets (×10^3^ μL) ^3^	326	325	12.1	0.92	0.89	0.97
Seric biochemistry						
Albumin (g/dL) ^3^	3.29	3.36	0.04	0.88	0.79	0.82
Total cholesterol (mg/dL) ^3^	133	133	1.02	0.95	0.95	0.97
Creatine kinase (U/L) ^3^	200 ^a^	254 ^b^	10.1	0.05	0.02	0.01
Cholinesterase (U/L) ^3^	161	149	3.05	0.07	0.05	0.03
Glucose (mg/dL) ^3^	62.0	60.5	1.25	0.94	0.92	0.91
Total protein (g/dL) ^3^	6.62	6.90	0.08	0.45	0.32	0.20
Ferritin (µg/L) ^3^	466	476	4.14	0.51	0.43	0.36
C-reactive protein (mg/dL) ^3^	9.99	9.96	0.07	0.98	0.97	0.98
Triglycerides (mg/dL) ^3^	12.2	14.6	0.79	0.62	0.50	0.48
Urea (mg/dL) ^3^	30.9	33.8	1.32	0.74	0.68	0.81
Globulin (g/dL) ^3^	3.33	3.54	0.06	0.09	0.06	0.05
IgG (mg/dL) ^3^	3.05 ^a^	3.55 ^b^	0.03	0.03	0.01	0.01
IgA (mg/dL) ^3^	3.47	3.45	0.02	0.96	0.92	0.89
Insulin (µU/mL) ^3^	7.12 ^b^	9.35 ^a^	0.46	0.05	0.11	0.13

^1^ Treatment effect when *p* < 0.05, with the difference between groups illustrated by different lowercase letters (a,b) on the same line. ^2^ Day effect and treatment × day interaction when *p* < 0.05, represented in Figure 2. ^3^ Corresponds to the effect of the treatment, represented by the average of the experimental period of 56 days.

**Table 5:** Oxidative status in blood serum of Jersey cows supplemented with organic chromium.

Variables	Control	Chromium	SEM	*p*: Treat ^1^
ROS (% of fluorescence intensity) ^3^	5.967	6.319	0.308	0.874
TBARS (nmol/mL) ^3^	10.62	10.77	0.465	0.971
MPO (µ moles of quinoneimine/30 min) ^3^	3.757	4.359	0.425	0.625
SOD (units/mg of protein) ^3^	0.169	0.289	0.012	0.262
SOD (units/mg of protein) ^2^				
d1	0.182	0.166	0.009	
d14	0.173	0.153	0.006	
d28	0.172	0.192	0.009	
d42	0.177 ^b^	0.324 ^a^	0.016	
d56	0.161 ^b^	0.322 ^a^	0.018	

^1^: There was no effect of treatment (*p* > 0.05). ^2^: There was a treatment × day interaction (*p* = 0.04) for SOD, where the difference between groups is illustrated by different lowercase letters (a, b) on the same line. ^3^ Corresponds to the effect of the treatment, represented by the average of the experimental period of 56 days.

## Data Availability

Data can be made available upon request.

## Supplementary Materials

The following supporting information can be downloaded at: https://www.mdpi.com/article/10.3390/ani15213111/s1, Table S1. Characteristics of the animals used in the research and information used to formulate the diets. Table S2. Ingredients and composition of diets provided in individual feeders (kg DM/cow/day).
