# Supplementary material for: Effects of Dietary Chromium Supplementation During Late Lactation on Productive Performance, Milk Composition, and Immune and Antioxidant Responses in Dairy Cows
Source: Animals (Basel). 2025 Oct 27;15(21):3111. doi: 10.3390/ani15213111 (PMC12607422; doi:10.3390/ani15213111)
Supplement: Supplementary file 1 [file animals-15-03111-s001.zip › animals-3902645-supplementary.pdf]

Table S1. Characteristics of the animals used in the research and information used to formulate the diets.

Herd	Animal characteristics
Race	Jersey
Animal category	Lactating cows
Age	60 months
Days in lactation	270
Days of gestation	180
Daily milk production	24.5 kg
Milk fat	4.7%
Protein in milk	3.44%

Table S1. Ingredients and composition of diets provided in individual feeders (kg DM/cow/day).

Ingredients	kg/cow/day
Corn silage, kg DM	8.68
Tifton hay-85, kg DM	1.58
Ground corn, kg DM	2.28
Soybean meal, kg DM	1.90
Wheat bran, kg DM	0.33
Soybean hulls, kg DM	0.54
Protected urea, kg DM	0.035
Sodium bicarbonate, kg DM	0.094
Dicalcium phosphate, kg DM	0.038
Calcitic limestone, kg DM	0.080
White salt, kg DM	0.055
Mineral supplement[1], kg DM	0.025

[1]Mineral supplement guarantee levels: Monensin 7500.00 mg/kg. Sulfur (min) 100.00 g/kg. Magnesium (min) 200.00 g/kg. Cobalt (min) 300.00 mg/kg. Copper (min) 5600.00 mg/kg. Iodine (min) 310.00 mg/kg. Manganese (min) 20.00 g/kg. Selenium (min) 160.00 mg/kg. Vitamin A (min) 2500000.00 IU/kg. Vitamin D3 (min) 750000.00 IU/kg. Vitamin E (min) 12500.00 IU/kg. Zinc (min) 23.75 g/kg. [2] PMR (partial mixed ration) is the feed provided in individual feeders, consisting of hay, concentrate, and corn silage.
